# Time to Treatment Failure as a Recorded Treatment-Process Measure in C-CAT. Reply to Comment on “Facility-Level Medical Oncology Specialist Availability and First-Line Time to Treatment Failure in Lung Cancer: A Nationwide C-CAT Registry Analysis. *Curr. Oncol.* 2026, *33*, 462”

**DOI:** 10.3390/curroncol33100632

**Published:** 2026-10-20

**Authors:** Shinya Kajiura, Hironaga Satake, Ryuji Hayashi, Takayuki Yoshino

**Affiliations:** 1 JSMO Secretariat Division, Japanese Society of Medical Oncology, Tokyo 105-0013, Japan; satakeh@kochi-u.ac.jp; 2 Department of Medical Oncology and Palliative Medicine, Toyama University Hospital, Toyama 930-0194, Japan; hsayaka@med.u-toyama.ac.jp; 3 Department of Medical Oncology, Kochi Medical School, Kochi University, Kochi 783-8505, Japan; 4 Board of Directors, Japanese Society of Medical Oncology, Tokyo 105-0013, Japan; 5 President, Japanese Society of Medical Oncology, Tokyo 105-0013, Japan; tyoshino@east.ncc.go.jp; 6 Department of Global Oncology, National Cancer Center Hospital East, Kashiwa 277-8577, Japan

**Keywords:** lung cancer, C-CAT, time to treatment failure, comprehensive genomic profiling, medical oncology, real-world data

## Abstract

We thank Karabuga and Kaya for their Comment on our nationwide C-CAT registry analysis of facility-level medical oncology specialist availability and first-line time to treatment failure (TTF) in lung cancer. We agree that the heterogeneous composition of TTF events and the predominantly post–first-line timing of C-CAT registration are important to interpretation. In this Reply, we clarify that TTF should be interpreted as the duration of a recorded first-line treatment process rather than as a direct measure of progression, treatment efficacy, survival benefit, facility quality, or individual specialist contribution. We also provide group-specific information on endpoint composition and the interval from first-line treatment initiation to C-CAT registration. Because treatment-ending causes were not clinically standardized and registration timing was itself post-treatment and selection-related, we did not perform additional cause-specific or registration-interval–stratified analyses. No new patient-level analysis was performed for this Reply.

We thank Karabuga and Kaya for their thoughtful Comment [1] on our nationwide C-CAT registry analysis [2]. We agree that the heterogeneous composition of time to treatment failure (TTF) and the predominantly post–first-line timing of C-CAT entry are important to interpretation. The original article acknowledged both issues and reported the relevant group-specific descriptive information in Supplementary Tables S8 and S10. We welcome the opportunity to make the resulting interpretive boundary more explicit.

In the original study, first-line TTF was defined from systemic therapy initiation to the recorded end of first-line treatment or death, whichever occurred first. It was therefore intended to represent the duration of a recorded first-line treatment process among patients who ultimately underwent comprehensive genomic profiling and entered C-CAT. Although TTF can be a pragmatic oncology endpoint and may correlate with progression-free survival in some trial settings [3], our TTF was not an imaging-based progression endpoint or a direct measure of treatment efficacy, survival benefit, facility quality, or individual specialist contribution. In the 5456-patient cohort, the fully adjusted complete-case estimate (*n* = 5188) for facilities with two or more versus zero or one registry-listed JSMO specialists was HR 0.911 (facility-cluster robust 95% CI, 0.806–1.029; *p* = 0.133): the point estimate was in the direction of longer recorded TTF, but the robust confidence interval included the null [2].

As reported in ref. [2] Supplementary Table S8, endpoint-source composition differed descriptively between the groups. Among 1477 patients in the zero-or-one-specialist group, 1336 (90.5%) had a recorded first-line end, 34 (2.3%) died without a recorded first-line end, and 107 (7.2%) were censored. The corresponding values among 3979 patients in the group with two or more specialists were 3344 (84.0%), 253 (6.4%), and 382 (9.6%). Termination reasons were available only for the 4680 recorded-end events; no reason was inferred for death-only events. Within that denominator, the major categories were “Invalidation cancellation” (48.1% vs. 51.6%), “Finished as planned” (31.2% vs. 21.9%), cancellation due to side effects (12.4% vs. 13.2%), and unknown, not recorded, or ambiguous reasons (4.8% vs. 9.6%) in the zero-or-one versus two-or-more groups, respectively. These contrasts show that the clinical composition of TTF cannot be assumed to be identical across groups; they do not, by themselves, indicate better or worse care.

We agree that cause-specific analyses would be informative if treatment-ending causes were clinically standardized and reliably ascertained. In the present C-CAT data, however, the largest category, “Invalidation cancellation,” is an exact source label without a validated mapping to radiographic progression or another standardized clinical cause. Moreover, death-only events had no termination reason, and 8.2% of recorded-end events had a reason that was unknown, not recorded, or ambiguous. Reclassifying these labels into clinical competing causes would therefore require unsupported assumptions. For this reason, we have not added a cause-specific Cox model; such a model could add apparent precision without improving clinical interpretability.

We also agree that the cohort was not an inception cohort of all patients starting first-line systemic therapy. C-CAT registration occurred after first-line initiation in 99.3% of the primary TTF cohort. Ref. [2] Supplementary Table S10 reported the timing by specialist-availability group: the median interval was 665 days (IQR, 359–1214), with 99.5% registered after first-line start, in the zero-or-one group, and 609 days (IQR, 308–1158), with 99.2% registered after first-line start, in the two-or-more group. These descriptive distributions should not be interpreted as demonstrating equivalence or excluding differential survivor, referral, or case-mix selection. Time-related bias is a recognized concern when cohort inclusion depends on events occurring after the chosen time origin [4].

The delayed-entry analysis reported in the original article was specifically a supportive overall survival sensitivity analysis, not a delayed-entry analysis of the primary TTF endpoint. For patients who ultimately entered C-CAT, delayed entry improved risk-set construction by excluding pre-registration time from observed at-risk time. It could not reconstruct a first-line inception cohort or recover patients who died, deteriorated, were not referred for comprehensive genomic profiling, or were never registered. We also did not stratify TTF by the registration interval because no prespecified clinically meaningful cutoff was available, the interval was itself post-treatment and selection-related, and such stratification would neither recover unregistered patients nor preserve the original estimand.

Accordingly, the study’s conclusion should remain bounded to patients who ultimately underwent comprehensive genomic profiling and entered C-CAT, and to the estimated association between a facility-level structural indicator and recorded first-line treatment-process duration. The Comment usefully reinforces this boundary. In parallel, JSMO and C-CAT have initiated discussions on optimizing future clinical data collection, including the prioritization of treatment-process variables that may improve the interpretability of subsequent registry-based analyses. Future national chemotherapy databases should identify patients at treatment initiation, capture standardized reasons for treatment discontinuation and line transitions, and include the referral denominator needed to evaluate how facility organization relates to cohort entry and subsequent treatment processes.

## Data Availability

No new data were created or analyzed in this Reply. The numerical information discussed is reported in the published article and its Supplementary Materials [2].
